# Evening Hymn

**Published:** 1862-10

**Authors:** 


### EVENING HYMN.

The day is past and gone,

The evening shades appear;

Oh ! may we all remember well,

The night of death is near.

Lord, keep us safe this night,

Secure from all our fears;

Beneath the pinions of thy love,

Till morning light appears.

And when we early rise,

To view the unwearied sun,

May we set out to win the prize,

And after glory run.

And when our days are past,

And we from time remove,

Oh ! may we in thy bosom rest—

The bosom of thy love.

### GOD IS LOVE.—1 John 4 : 8.

For God so loved the world, that he gave his only begotten Son, that whosoever believeth in him should not perish, but have everlasting life.—John 3 : 16.

Ye are my friends, if ye do whatsoever I command you.—John 15 : 14.

Come unto me, all ye that labor and are heavy laden, and I will give you rest.—Matt. 11 : 28.

And him that cometh to me, I will in no wise cast out.—John 6 : 37.

For what shall it profit a man, if he shall gain the whole world, and lose his own soul ? Or what shall a man give in exchange for his soul ?—Mark 8 : 36, 37.

Be sober, be vigilant, casting all your care upon him, for he careth for you.—1 Peter 5 : 8, 7.

This is a faithful saying, and worthy of all acceptation, that Christ Jesus came into the world to save sinners, of whom I am chief.—1 Timothy 1 : 15.

And Jesus said unto him, Verily I say unto thee, To-day shalt thou be with me in Paradise.—Luke 23 : 43.

Life is the time to serve the Lord,
The time t' insure the great reward;
And while the lamp holds out to burn,
The vilest sinner may return.

Our Father which art in heaven, hallowed be thy name. Thy kingdom come. Thy will be done in earth, as it is in heaven. Give us this day our daily bread. And forgive us our debts, as we forgive our debtors. And lead us not into temptation, but deliver us from evil. For thine is the kingdom, and the power, and the glory, forever. Amen.—Matt. 6 : 9–13.

